# Persistence of Natural Killer (NK) cell lymphocytosis with hyposplenism without development of leukaemia

**DOI:** 10.1186/1472-6890-5-8

**Published:** 2005-09-07

**Authors:** Sujoy Khan, K Myers

**Affiliations:** 1 Department of Haematology, Prince Charles Hospital, Merthyr Tydfil, Wales, CF47 9DT UK; 2 Department of Immunopathology, St. Bartholomew's Hospital, 51-53 Bartholomew's Close, West Smithfield, London, EC1A 7BE UK

## Abstract

**Background:**

Natural killer (NK) cell lymphocytosis usually has an indolent course and can progress into massive lymphocytosis with development of cytopenias and neoplastic diseases. NK-cells usually express one or more "NK-associated" antigens (CD16, CD56, CD57). Reactive expansions are seen in autoimmune diseases, viral infections, solid tumours and non-Hodgkin's lymphoma.

**Case presentation:**

We report a lady with a benign clinical course over 10 years and persistent CD8+/CD3-/CD57+/CD16+ LGL proliferation with presence of Howell-Jolly bodies (functional hyposplenism), an association not previously described.

**Conclusion:**

We discuss the possible causes of clonal expansion and conclude that this may be part of the spectrum of immune dysregulation associated with NK-cell lymphocytosis.

## Background

The lymphoproliferative disease of granular lymphocytes (LDGL) results from the chronic proliferation of large granular lymphocytes (LGL) that may result from antigenic stimulation^1,2^. Natural Killer (NK) cells constitute approximately 15% of the peripheral blood mononuclear cell fraction. NK cells lack both CD3 and T-cell receptor expression, majority express CD56 and/or CD16 (F_cγ _receptor). Granular lymphocytosis greater than 2,000/μL lasting for more than 6 months is regarded as the criteria to define the disease [1,2]. Patients with chronic, indolent NK lymphocytosis may develop cytopenias, splenomegaly, vasculitic skin lesions, and peripheral neuropathy [3]. We discuss a unique case of chronic, indolent NK lymphocytosis who presented with severe hyposplenism and has not developed leukaemia over a decade.

## Case presentation

A 46-year-old lady was referred to the haematology clinic for evaluation of lymphocytosis in May 1993. She had severe lethargy and intermittent right upper abdominal discomfort without any significant loss in weight. Her past medical history included essential hypertension controlled on atenolol 100 mg once daily and was also on frusemide 40 mg once daily. She had no significant surgical history other than having undergone cholecystectomy in 1972. She had never smoked nor consumed alcohol. Physical examination showed no evidence of lymphadenopathy. Complete blood count showed normal haemoglobin concentration 14.8 g/dl, macrocytosis (MCV 100.1), raised white cell count at 13.4 × 10^9^/L, lymphocytosis (absolute number 6.3 × 10^9^/L), and normal neutrophil count (absolute number 5.6 × 10^9^/L). Peripheral blood showed numerous Howell-Jolly bodies within erythrocytes. Thyroid function tests, protein electrophoresis, C-reactive protein, immunoglobulin levels and autoimmune screening were normal. Ultrasonography and computed tomography scan of the abdomen and pelvis did not reveal retroperitoneal or mediastinal lymphadenopathy, but the spleen was noted to be very atrophic. Gastroscopy showed multiple gastric erosions and the initial impression was of celiac disease complicated by lymphoma and lymphocytosis.

Duodenal biopsy showed well-formed villi and no increase in intraepithelial lymphocytes thereby excluding celiac disease. Colonoscopy and barium studies were normal. She was clinically diagnosed to have functional hyposplenism, considering the presence of Howell-Jolly bodies, and was given hemophilus influenzae (HiB) vaccine, pneumovax vaccine and counselled for long term oral Penicillin V. In November 1994, another complete blood count showed a white cell count of 15.3 × 10^9^/L with absolute number of lymphocytes of 6.6 × 10^9^/L. Bone marrow biopsy showed 25% infiltration with large granular lymphocytes. Peripheral blood smear revealed that the majority of lymphocytes had atypical morphology with large atypical nuclei and abundant cytoplasm containing fine azurophilic granules. Immunophenotyping showed CD16+/CD3- cells which were mainly reactive and another clonal cells which were CD57+/CD8+ suggesting NK-LGL (Natural Killer-Large Granular Lymphocyte) activity. 80% CD3+ cells were α/β and 20% γ/δ and a clonal population of CD16+/CD56+ cells.

She presented in March 1995 with lethargy and diffuse enlargement of her thyroid gland. Lymphocytosis persisted (WBC 15.7 × 10^9^/L, absolute lymphocyte count 8.2 × 10^9^/L), antimicrosomal antibody was positive at a titre of 1:6400, anti thyroglobulin antibody was positive at 1:1280 but thyroid function tests (TSH 1.69, fT_4 _13.0) and isotope thyroid scan I^123 ^were normal. Immunoglobulins, C3, C4 and C-reactive protein levels were normal. There was spontaneous regression of her thyroid swelling by December 1996 at which time antimicrosomal antibody was negative. Follow-up for 10 years showed the persistence of lymphocytosis (February 2004-WBC 12.5 × 10^9^/L, absolute lymphocyte count 5.4 × 10^9^/L) without development of autoimmune disease, autoantibodies, neutropenia or infections.

## Conclusion

This is the first report of the unusual occurrence of Howell-Jolly bodies in a patient with persistent NK-LGL lymphocytosis. She did not develop autoimmune disease, neutropenic infections or vasculitic syndromes but the lymphocytosis persisted.

Cytogenetic, molecular analyses and monoclonal antibodies (MoAbs) both against the V-gene regions of the T-cell receptor (TCR) and the molecules of the p58 family on NK cells [4] are used to characterize the expanded cells. Polyclonal proliferation may represent a preneoplastic condition, since there is some evidence that suggests that it is probably a multistep process [5]. In such cases, the clonal expansion might evolve from the abnormal immunoregulation of a response to inciting antigens, for example, viruses [5].

The Yorkshire Leukaemia Group investigated 870 adults with 'persistent' LGL/NKa^+ ^(Large Granular Lymphocyte/Natural Killer associated) expansions suggest that clonal expansions may be more frequent than reported and found high proportion with persistent neutropenia and all patients with CD8+NKa+ abnormalities had rearranged TCR genes [6]. Splenomegaly has been reported in 19 % in a case series of 68 patients with clonal T-LGL proliferations [7], but uniquely, in our case, the lymphocytosis has remained stable and Howell-Jolly bodies were observed. The presence of Howell-Jolly bodies and liver-spleen scintigraphy showing absence of spleen is considered diagnostic of functional hyposplenia [8]. An autoimmune mechanism seems probable but not certain [9].

Clonality does not imply malignancy. It has been detected during autoimmune processes, including rheumatoid arthritis, and in bone marrow transplantation recipients [9]. Clonal populations in such cases most likely represent epiphenomenon of an immunoregulatory disorder. There is evidence of persistent CD8+ clonal expansions in normal elderly individuals [10] and clonal V-alpha 12.1^+ ^T-cell expansions in uncomplicated rheumatoid arthritis [11] that could mean that these proliferations are benign.

In summary, our lady with persistent NK cell lymphocytosis and hyposplenism had a benign clinical course over a decade. We report this case as presence of Howell-Jolly bodies has not been previously reported and postulate that the hyposplenism is probably a part of the spectrum of immune dysregulation associated with NK-LGL.

## Abbreviations

LGL-large granular lymphocytes; NK-Natural Killer; TCR-T-cell receptor.

## Competing interests

The author(s) declare that they have no competing interests.

## Authors' contributions

SK drafted the initial manuscript. KM made changes to the manuscript and as corresponding author had full access to all data and was responsible for the decision to submit for publication.

## Pre-publication history

The pre-publication history for this paper can be accessed here:


